# Re-Validation of the Van Rie HIV/AIDS-Related Stigma Scale for Use with People Living with HIV in the United States

**DOI:** 10.1371/journal.pone.0118836

**Published:** 2015-03-04

**Authors:** Aaron M. Kipp, Carolyn M. Audet, Valerie A. Earnshaw, Jared Owens, Catherine C. McGowan, Kenneth A. Wallston

**Affiliations:** 1 Division of Epidemiology, Vanderbilt University Medical Center, Nashville, Tennessee, United States of America; 2 Vanderbilt Institute for Global Health, Vanderbilt University Medical Center, Nashville, Tennessee, United States of America; 3 Department of Preventive Medicine, Vanderbilt University Medical Center, Nashville, Tennessee, United States of America; 4 Center for Interdisciplinary Research on AIDS, Yale University, New Haven, Connecticut, United States of America; 5 Department of Psychology, Middle Tennessee State University, Murfreesboro, Tennessee, United States of America; 6 Division of Infectious Diseases, Vanderbilt University Medical Center, Nashville, Tennessee, United States of America; 7 Vanderbilt University, School of Nursing, Nashville, Tennessee, United States of America; Johns Hopkins School of Public Health, UNITED STATES

## Abstract

There is little consensus about which of the many validated human immunodeficiency virus (HIV) stigma scales should be regularly used, with few being re-validated in different contexts or evaluated for how they compare to other, existing HIV stigma scales. The purpose of this exploratory study was to re-validate the Van Rie HIV/AIDS-Related Stigma Scale, originally validated in Thailand and using a third-person wording structure, for use with people living with HIV in the United States. Adult HIV clinic patients completed a survey including the Berger and Van Rie scales, and measures of social support and depression. Eighty-five of 211 (40%) eligible participants provided data for both stigma scales. Exploratory factor analyses identified three factors to the Van Rie scale: Loss of Social Relationships (new subscale), Managing HIV Concealment (new subscale), and Perceived Community Stigma (original subscale). These subscales were moderately inter-related (r = 0.51 to 0.58) with acceptable to excellent reliability (Cronbach’s alpha = 0.69 to 0.90). The Van Rie subscales were also moderately inter-correlated with the Berger subscales (r = 0.44 to 0.76), had similar construct validity, and tended to have higher mean stigma scores when compared with Berger subscales that were conceptually most similar. The revised Van Rie HIV-related Stigma Scale demonstrates good validity and internal consistency, offering a valid measure of HIV stigma with a three-factor structure. The third-person wording may be particularly suitable for measuring stigmatizing attitudes during an individual’s transition from at-risk and undergoing HIV testing to newly diagnosed, a time when experiences of discrimination and processing issues of disclosure have not yet occurred. The stigma mechanisms for individuals making this transition have not been well explored. These scenarios, combined with the observed non-response to the Berger Enacted Stigma subscale items (a surprise finding), highlight gaps in our understanding of HIV stigma and how best to measure it.

## INTRODUCTION

More than 30 years into the HIV epidemic, HIV-related stigma continues to be a concern for people living with HIV (PLHIV), their families, and caregivers [1,2]. The effort to quantify the presence and impact of HIV stigma has resulted in the development of numerous scales measuring HIV stigma. Literature reviews [3,4] and a search of recent scale development publications identified at least 34 formally developed HIV stigma scales, with nearly equal proportions developed in the United States (US) and internationally, measuring HIV stigma among PLHIV, uninfected community members, and medical providers. Nevertheless, new HIV stigma scales continue to be developed [5–9] and there appears to be no consensus on which measures should be regularly used by researchers or other personnel. To our knowledge, few scales have been validated in multiple contexts (see for example the Internalized AIDS-related Stigma Scale [10,11], the HIV/AIDS Stigma Instrument—PLWA [12,13], Genberg et al. [14], Nyblade et al. [15], and the Berger HIV Stigma Scale described below). While it is difficult to systematically track the use of every validated stigma measure, similar concerns have been expressed in systematic reviews of stigma scales used in HIV treatment adherence studies [16] and HIV stigma interventions [17]. These reviews found numerous different scales being used, many of which had not been validated. And even among validated scales, few were used in multiple studies, with the Berger HIV Stigma Scale being the primary exception. An important contribution, therefore, would be to compare and further refine and validate existing measures of HIV stigma, rather than develop new measures. Unfortunately, even recently developed scales lack any comparison with an existing HIV stigma scale [5–8].

The purpose of this exploratory study was to re-validate the Van Rie HIV/AIDS-Related Stigma Scale (hereafter referred to as the Van Rie scale), originally developed in Thailand among tuberculosis (TB) and TB/HIV patients [18,19], for use among PLHIV in the US. A secondary purpose was to compare it with the Berger HIV Stigma Scale (hereafter referred to as the Berger scale) [20], which is more widely used and has undergone extensive re-validation both internationally [21–23] and in the US [24,25]. In so doing, this study will help to better understand how existing stigma scales compare with each other.

The Van Rie scale was chosen for a variety of reasons. First, it uses a third-person wording structure for experiences of HIV stigma (e.g., “People with HIV lose friends when they share with them they have HIV”) in contrast to the first-person wording used in nearly all HIV stigma scales, including the Berger scale (e.g., “I have lost friends by telling them that I have HIV“). During the original development in Thailand, this was felt to be culturally more appropriate for obtaining more valid responses. Outside of that context, the third-person wording may still be preferred when measuring stigma among newly diagnosed individuals, a time when stigmatizing experiences (e.g., discriminations) have not yet occurred. The Van Rie scale therefore offers a complimentary, but different, way of measuring HIV stigma than the Berger scale. Second, it was developed in parallel with a TB stigma scale with common wording across both scales. The ability to measure stigma across different conditions is rare, with scales typically being condition specific, though some exceptions exist [26–28]. There have been recent calls for condition *non-specific* measures given findings that stigma manifestations, consequences, and even item wording are shared across conditions [29]. Such scales would help in comparing levels and consequences of stigma among people with different conditions and a better understanding of health-related stigma in general [28]. Finally, the Van Rie scale was developed outside of the US; therefore validation in a US population will further inform its cross-cultural application, as has occurred with the Berger scale and only a few others [10,14,15].

## METHODS

### Ethics statement

This research was approved by the Vanderbilt Institutional Review Board (IRB# 110829) and all participants provided signed informed consent.

### Participants and Procedures

The study was conducted between November 2011 and January 2012 at the Vanderbilt University Medical Center outpatient HIV clinic in Nashville, Tennessee. The clinic provides medical, psychiatric, and nutritional care to an estimated 80% of PLHIV living in the central portion of Tennessee. In 2010, more than 2,800 PLHIV were seen by a provider at the clinic.

Adult (≥ 18 years of age) PLHIV with at least one prior medical provider visit at the clinic were eligible to participate if they were able to communicate in English and capable of providing informed consent. Eligible patients were approached by research staff working specifically on this project (co-authors AMK or JO) while waiting in the exam room to be seen by the provider. Interested patients met with the same research staff in a private interview room following their clinic appointment to complete the 30-minute interviewer-administered questionnaire. Upon completion, participants were compensated for their time with a gift card to a retail store.

### Measures

In addition to the psychosocial measures listed below, the questionnaire collected information on socio-demographics, HIV risk category, how long participants have been (a) living with HIV, (b) attending the clinic, and (c) receiving antiretroviral therapy, and to whom they had disclosed their HIV status.

#### HIV stigma

Among PLHIV, one or more of the following three stigma domains are typically measured: *Enacted stigma*, defined as experiences of discrimination because of HIV; *anticipated stigma*, defined as the belief that one will experience prejudice or discrimination in the future as a result of HIV; and *internalized stigma*, defined as internalizing and endorsing the negative beliefs and feelings about PLHIV that are held by others [4]. The two HIV stigma scales used in this study measured these domains in different ways. The first was a shortened version of the Berger HIV Stigma Scale [20] validated by Bunn et al., which contains four subscales (domains): Enacted Stigma, Disclosure Concerns (a form of anticipated stigma), Negative Self-Image (e.g. internalized stigma), and Concern with Public Attitudes towards HIV (e.g., anticipated stigma) [24]. The second was the Van Rie scale, which originally contained two subscales: Patient Felt/Experienced Stigma (a mix of items expressing enacted and internalized stigma) and Perceived Community Stigma (e.g., anticipated stigma) [18,19]. Data from an earlier qualitative study [30] suggested that the original item stem “Some people” may affect validity by unnecessarily inflating scores. Therefore the stem was modified to “People…” for this study. Additionally, one item from the Felt/Experienced Stigma subscale was excluded *a priori* from factor analyses because cognitive interviews indicated it was a poorly worded and confusing item. For both stigma scales, participants responded to each item using a 4-point Likert response scale of “strongly disagree,” “disagree,” “agree,” and “strongly agree”. Subscale and total stigma scores were created by averaging item responses, which creates scores ranging from 0 to 3, allowing comparison across scales with different numbers of items. Higher scores indicate higher stigma levels.

#### Depression

Thirteen items from the Center for Epidemiologic Studies—Depression (CES-D) scale [31] were used to measure depressive mood, excluding seven items measuring the somatic component of depression [32]. Participants indicated how often during the previous week they felt or experienced each item using a 4-point response scale: “Rarely or None (<1 day),” “Some or a little (1–2 days),” “Occasional or moderate (3–4 days),” or “Most or all the time (5–7 days).” Responses were summed resulting in a score ranging from 0 to 39, with higher scores indicating higher levels of depressed mood. Cronbach’s alpha for this 13-item scale was 0.88.

#### Social Support

Nineteen items from the O’Brien social support scale [33] were used to measure three domains: Perceived Availability of Support, Social Conflict, and Objective Social Integration. Participants responded to the seven Perceived Availability of Support items using a 4-point response scale of “Definitely not,” “Probably not,” “Probably yes,” and “Definitely yes,” and to the six Social Conflict items and six Subjective Social Integration items using a 5-point response scale of “Never,” “Rarely,” “Sometimes,” “Frequently,” and “All of the time.” Responses for each subscale were summed resulting in scores ranging from 0 to 21, 0 to 24, and 0 to 24 for perceived social support, social conflict, and social integration, respectively, with higher scores indicating higher levels of each domain. A total social support score was developed by reverse coding the social conflict scores and summing items across all subscales, resulting in a score ranging from 0 to 69. Cronbach’s alpha for the Perceived Availability of Support, Social Conflict, Subjective Social Integration, and Total Social Support scales were 0.89, 0.84, 0.82, and 0.91, respectively.

#### Social Desirability

A short form of the Marlowe-Crowne Social Desirability Scale [34] was used to measure potential bias due to social desirability. This index uses 10 items with a dichotomous response option of “True or mostly true” and “False or mostly false”. Responses were summed resulting in a score ranging from 0 to 10, with higher scores indicating a greater tendency to respond in a socially desirable manner.

For all of the psychosocial measures, additional response options of “not applicable” and “refused” were available to assure participants that they were free to not answer items. Distinguishing between refusals and items deemed “not applicable” was intentional out of concern that some items incorrectly assumed a participant had certain knowledge or experiences. When participants chose not to respond to an item, they almost exclusively reported “not applicable.” For analysis purposes, any response of “not applicable” or “refused” was coded as a missing response for that item.

### Analysis

#### Factor analyses

Missing item responses were imputed with the mean of the non-missing items from the corresponding subscale if at least 70% of the items were non-missing. If item completeness for any subscale was <70%, the participant was excluded from the factor analysis. Exploratory factor analyses using principal components with oblique (Promax) rotation and Kaiser normalization were conducted separately for the Berger and Van Rie scales. The sample size was not sufficient to conduct a confirmatory factor analysis on either measure and it was felt that an exploratory factor analysis was warranted given the new population for the Van Rie scale (US PLHIV instead of Thai TB/HIV patients) and uncertainty to the number of factors that would emerge. We anticipated that one or both of the original subscales may be split into smaller subscales and/or items may be dropped entirely from a subscale. However, it was decided *a priori* that no items from one of the original subscales would be combined with items from the other into a new subscale. The rationale for this was two-fold. First, the original two subscales on the Van Rie scale each had uniquely different item word stems (“People who have HIV…” vs. “People [in general]…”), reflecting an important difference between who is being stigmatized versus who is the stigmatizer. Second, as previously stated, the third-person wording “People with HIV…” was developed specifically as a proxy for personal experiences with HIV (e.g., “People with HIV lose friends when they share with them they have HIV” versus “I have lost friends by telling them that I have HIV“). The subscale or subscales utilizing the item stem “People with HIV…” are intended to be comparable to the first-person wording used in nearly all other HIV stigma scales. Thus, combining items from “People [in general…” with items from “People with HIV…” into the same subscale was not deemed to be appropriate, much like we would not consider combining third-person items from the Berger scale with first-person items in a new subscale (e.g., “Most people are uncomfortable around someone with HIV” with “I am hurt by how people reacted to learning I have HIV”). In contrast to the Van Rie scale, the Berger scale was expected to retain its four factor structure given its extensive use and re-validation so attention was only paid to whether items loaded on their expected factors. For both stigma scales, Items with factor loadings ≥0.40 were retained if they met the preceding criteria. Items were dropped if factor loadings were <0.40 or were ≥0.40 for more than one factor.

#### Reliability and validity

After identifying a revised set of subscales and items, internal consistency (how well a set of items conceptually fit together) was assessed by calculating Cronbach’s alpha. Concurrent validity (the degree to which the construct being measured correlates with another measure of the same construct) was assessed by calculating Pearson correlations between the two stigma scales. Convergent validity (the degree to which the construct being measured correlates with a related construct) was assessed by correlating the stigma scales with the theoretically-related constructs of depressed mood and social support used in the original development of these scales [18,20,35]. Discriminant validity (the degree to which the construct being measured does not correlate with an unrelated construct) was determined by correlating the stigma scales with the social desirability index.

All analyses were conducted using Stata/SE 12 (StataCorp, College Station, TX).

## RESULTS

Two hundred and eleven patients were invited to participate, of whom 85 (40%) completed a questionnaire. Table 1 shows the distribution of participant characteristics, which are comparable to those of the larger clinic patient population [36,37]. The proportion of participants responding to all items on each original subscale ranged from 73% to 98%. The Berger Disclosure Concerns and Negative Self-Image subscales had nearly complete responses to all items (Table 2) while the Berger Enacted Stigma subscale had item non-response of at least 5% on nine of the original 11 items and subsequently the lowest proportion of complete responses (72.9% [95% CI: 63.3–82.6%]), with a confidence interval that excluded the proportion of complete responses observed for all other subscales. The Van Rie Perceived Community Stigma (83.5%; 95% CI: 75.5–91.6%) and Berger Concern with Public Attitudes (87.1%; 95% CI: 79.8–94.3%) subscales had similar, improved proportions of complete responses, but were still lower than the remaining subscales.

**Table 1:** Socio-demographic and clinical characteristics of study participants (n = 85).

Category	Subcategory	Distribution
Age (median; IQR)		45 (19)
Gender (N; %)	Male	63 (74.1)
	Female	22 (25.9)
Race/ethnicity (N; %)	Non-Hispanic White	43 (50.6)
	Non-Hispanic Black	35 (41.2)
	Hispanic	5 (5.9)
	Non-Hispanic Other	2 (2.4)
	Missing	0 (0.0)
Foreign birth (N; %)	No	4 (4.7)
	Yes	81 (95.3)
Education (N; %)	≤High School	38 (44.7)
	>High School	47 (55.3)
	Missing	0 (0.0)
HIV risk (N; %)^a^	Heterosexual	25 (29.4)
	MSM	43 (50.6)
	IDU	5 (5.9)
	Other/Unknown	12 (14.1)
Years since HIV diagnosis (median; IQR)		10 (12)
Disclosed to 3 or 4 of the following: partner, parent, other family, friend (N; %)	No	29 (34.1)
	Yes	56 (65.9)
Taking antiretroviral treatment (N; %)	No	9 (10.6)
	Yes	76 (89.4)
	Unknown	0 (0.0)

^a^ MSM, men who have sex with men; IDU, injection drug user.

**Table 2:** Distribution of missing responses to individual items among all study participants prior to exploratory factor analysis (n = 85).

	Berger HIV Stigma Scale				Van Rie HIV-related Stigma Scale	
	Enacted stigma	Disclosure concerns	Negative self-image	Concern with public attitudes	Felt/ experienced stigma^b^	Perceived community stigma
	N (%)	N (%)	N (%)	N (%)	N (%)	N (%)
Item 1	8 (9.4)	0 (0.0)	0 (0.0)	0 (0.0)^a^	0 (0.0)	4 (4.7)^a^
Item 2	7 (8.2)	1 (1.2)	0 (0.0)	2 (2.4)	1 (1.2)	8 (9.4)
Item 3	5 (5.9)	0 (0.0)	0 (0.0)	10 (11.8)	0 (0.0)	1 (1.2)
Item 4	4 (4.7)	2 (2.4)	1 (1.2)	0 (0.0)^a^	1 (1.2)	3 (3.5)
Item 5	9 (10.6)	0 (0.0)	0 (0.0)	0 (0.0)	0 (0.0)^c^	1 (1.2)^a^
Item 6	6 (7.1)	0 (0.0)	1 (1.2)	1 (1.2)	0 (0.0)	1 (1.2)
Item 7	11 (12.9)	0 (0.0)			0 (0.0)	2 (2.4)
Item 8	6 (7.1)				1 (1.2)^a^	0 (0.0)
Item 9	2 (2.4)^a^				4 (4.7)^a^	0 (0.0)
Item 10	6 (7.1)^a^				0 (0.0)	5 (5.9)
Item 11	5 (5.9)^a^					2 (2.4)^a^
Complete responses to all items	62 (72.9)	83 (97.7)	83 (97.7)	74 (87.1)	80 (94.1)	71 (83.5)
Missing 1 item only	10 (11.8)	1 (1.2)	2 (2.4)	10 (11.8)	4 (4.7)	7 (8.2)
Missing 2 items	2 (2.4)	1 (1.2)				5 (5.9)
Missing 3 items	1 (1.2)			1 (1.2)	1 (1.2)	1 (1.2)
Missing 4+ items	10 (11.8)					1 (1.2)

^a^ item excluded from revised set of items following exploratory factor analysis.

^b^ The original version of this sub-scale contained 10 items and has was termed the “Patient perspective” or “Felt/Experienced stigma” sub-scale.

^c^ item excluded a priori before exploratory factor analysis (see Methods).

### Exploratory factor analyses

Four factors with eigenvalues >1.0 were identified for the Van Rie scale (Table 3). The fourth factor was dropped from further consideration because the two items were not conceptually related and two was deemed too few items to warrant a separate factor. The remaining three factors accounted for 57% of the total variance in the overall Van Rie scale. The first factor was consistent with the original Perceived Community Stigma subscale. Three items were dropped from the original 11 items: two which loaded on a different factor that contained three items from the original Felt/Experienced Stigma subscale, and one loaded on multiple factors. The revised set of eight Perceived Community Stigma items had factor loadings ranging from 0.63 to 0.91.

**Table 3:** Eigenvalues and rotated factor loadings for the Van Rie HIV-related Stigma Scale items^a^.

	Factor 1	Factor 2	Factor 3	Factor 4
Factor eigenvalues	8.3	1.7	1.3	1.1
**Felt/Experienced stigma subscale items**				
People who have HIV lose friends when they share with them they have HIV (4)		0.59		
People who have HIV feel alone (2)		0.54		
People who have HIV feel hurt because of how others react to knowing they have HIV (1)		0.43		
People who have HIV are afraid that other people in the community will talk about them having HIV (3)			0.75	
People who have HIV will choose carefully who they tell about having HIV (10)			0.73	
People who have HIV worry that others will reveal their secret (6)			0.68	
People who have HIV try very hard to keep their HIV a secret (7)			0.57	
People who have HIV keep their distance from others to avoid spreading the HIV virus (8)^b^				0.84
People who have HIV People who have HIV feel guilty because their family has the burden of caring for them (9)^b^				0.48
People who have HIV are afraid to tell those outside their family that they have HIV (5)^b^	excluded *a priori*			
**Perceived community stigma items**				
People are afraid of those with HIV (8)	0.91			
People do not want to talk to others with HIV (4)	0.75			
People prefer not to have those with HIV living in their community (10)	0.67			
People think that people with HIV are unclean (9)	0.61			
People try not to touch others with HIV (7)	0.61			
People feel uncomfortable being near those with HIV (3)	0.60			
People do not want those with HIV playing with their children (2)	0.58			
If a person has HIV, community members will behave differently towards that person for the rest of his or her life (6)	0.63			
People keep their distance from those with HIV (5)^b^	0.50		0.46	
People think that those with HIV are disgusting (1)^b^		0.83		
People think that people with HIV get what they deserve (11)^b^		0.63		

^a^ Blanks represent factor loadings <0.40; number in parenthesis corresponds to item number in Table 2.

^b^ item excluded from final set of items.

The original Van Rie Felt/Experienced Stigma subscale was split into two factors comprising two new subscales (Table 3). The first subscale contained three items with factor loadings ranging from 0.43 to 0.59 that reflected loss of social relationships. Another subscale contained four items with factor loadings ranging from 0.57 to 0.75 that reflected concerns about managing HIV concealment or disclosure.

The Berger scale retained its four factor structure with few changes in where items loaded (Table 4). The four factors accounted for 65% of the total variance of the overall scale. Three of the 11 items of the Enacted Stigma subscale were dropped because they did not load on the expected factor, with the remaining eight items having factor loadings that ranged from 0.48 to 0.98. Two of the six items from the Concern with Public Attitudes subscale also did not load on the expected factor, with the remaining four items having factor loadings that ranged from 0.52 to 0.79. All items for the remaining two subscales loaded on their expected factors: Disclosure Concerns (7 items) had factor loadings ranging from 0.50 to 0.90 and Negative Self-Image (6 items) had loadings ranging from 0.76 to 0.92.

**Table 4:** Eigenvalues and rotated factor loadings for the Berger HIV Stigma Scale items^a^.

	Factor 1	Factor 2	Factor 3	Factor 4	Factor 5
Factor eigenvalues	12.1	3.4	2.6	1.4	1.2
**Enacted stigma items**					
I have lost friends by telling them that I have HIV (1)	0.98				
People have physically backed away from me because I have HIV (7)	0.79				
I have stopped socializing with some people due to their reactions towards HIV (4)	0.77				
I am hurt by how people reacted to learning I have HIV (2)	0.71				
Some people who know that I have HIV have grown more distant (8)	0.71				
People I care about stopped calling me after learning that I have HIV (5)	0.66				
People seem afraid of me because I have HIV (6)	0.48				
People avoid touching me if they know I have HIV (3)	0.48				
People who know that I have HIV ignore my good points (9)^b^			0.53		
People don’t want me around their children once they know that I have HIV (10)^b^	0.41		0.53		
I regret having told some people that I have HIV (11)^b^					
**Disclosure concerns items**					
I am very careful who I tell that I have HIV (2)				0.90	
Telling someone I have HIV is risky (6)				0.83	
I work hard to keep my HIV a secret (3)				0.79	
In many areas of my life, no one knows that I have HIV (5)				0.74	
I worry that people may judge me when they learn that I have HIV (7)				0.69	
I have told people close to me to keep my HIV a secret (4)				0.60	
I worry people who know I have HIV will tell others (1)				0.50	
**Negative self-image items**					
Having HIV makes me feel unclean (3)		0.92			
I feel I’m not as good as others because I have HIV (2)		0.87			
Having HIV makes me feel that I’m a bad person (1)		0.80			
I feel guilty because I have HIV (6)		0.78			
Having HIV is disgusting to me (4)		0.77			
People’s attitudes about HIV make me feel worse about myself (5)		0.76			
**Concern with public attitudes items**					
People with HIV are treated like outcasts (6)			0.79		
Most people are uncomfortable around someone with HIV (5)			0.70		
Most people with HIV lose their jobs when employers learn that they have HIV (3)			0.67		
Most people with HIV are rejected when others learn that they have HIV (2)			0.52		
Most people believe a person who has HIV is dirty (4)^b^					0.71
Most people think that a person with HIV is disgusting (1)^b^					0.69

^a^ Blanks represent factor loadings <0.40: number in parenthesis corresponds to item number in Table 2.

^b^ item excluded from final set of items.

### HIV stigma scores

Stigma, depression, and social support scores were normally or approximately normally distributed. Table 5 shows the number of items, Cronbach’s alpha, mean score, and completeness of responses for the revised HIV stigma scales and the other measures. Completeness of responses to subscale items improved slightly for the Van Rie Perceived Community Stigma (87% with complete responses) and Berger Enacted Stigma (79%) subscales with the exclusion of three items from each, but was still low relative to the other subscales. Mean stigma scores ranged from 1.19 for the Berger Negative Self-Image subscale to 2.24 for the Van Rie Managing HIV Concealment subscale (possible range of 0 to 3). Stigma scores for the two new Van Rie subscales were generally higher than the Berger subscales, while scores were nearly identical for the Van Rie Perceived Community Stigma and Berger Concern with Public Attitudes subscales (1.70 vs. 1.74, respectively), both of which measure anticipated stigma by asking about how people (in general) think of, feel about, or act towards PLHIV.

**Table 5:** Mean scores and other scale characteristics for HIV stigma, depression, social support and social desirability (n = 85).

Category	Subcategory	No. Items (score range)	Cronbach’s alpha	Mean (SD)	% Complete responses
Van Rie HIV stigma	Loss of Social Relationships	3 (0 to 3)	0.69	1.93 (0.61)	99%
	Managing HIV Concealment	4 (0 to 3	0.74	2.24 (0.47)	100%
	Perceived Community stigma	8 (0 to 3)	0.90	1.70 (0.52)	87%
	Total stigma	15 (0 to3)	0.90	1.90 (0.45)	86%
Berger HIV stigma	Enacted stigma	8 (0 to 3)	0.90	1.37 (0.64)	79%
	Disclosure concerns	7 (0 to 3)	0.91	1.97 (0.63)	98%
	Negative self-image	6 (0 to 3)	0.93	1.19 (0.66)	98%
	Concern with public attitudes	4 (0 to 3)	0.88	1.74 (0.63)	87%
	Total stigma	25 (0 to 3)	0.94	1.53 (0.50)	73%
Depression		13 (0 to 39)	0.88	9.74 (8.26)	99%
Social Support	Perceived availability of support	7 (0 to 21)	0.89	17.98 (4.17)	100%
	Subjective social integration	6 (0 to 24)	0.82	15.38 (5.86)	100%
	Conflict	6 (0 to 24)	0.84	8.61 (5.05)	100%
	Total social support	19 (0 to 69)	0.91	48.74 (12.98)	100%
Social Desirability		10 (0 to 10)	0.59	7.19 (1.93)	100%

### Correlations with other stigma and related constructs

Correlations among the revised HIV stigma subscales are shown in Table 6. The three Van Rie subscales were moderately inter-correlated with each other, while the four Berger subscales were low to moderately inter-correlated. When comparing the two stigma scales, the total scores were highly correlated (r = 0.80), but the individual subscales were less strongly inter-correlated (r = 0.44 to 0.76). The Van Rie Loss of Social Relationships subscale was inter-correlated with both the Berger Enacted Stigma and Concern with Public Attitudes subscales. The Van Rie Managing HIV Concealment subscale had the highest correlation with the Berger Disclosure Concerns subscale, and the Van Rie Perceived Community Stigma subscale had the highest correlation with the Berger Concern with Public Attitudes subscale. All of the Van Rie subscales had generally lower correlations with the Berger Negative Self-Image subscale.

**Table 6:** Correlation coefficients between HIV stigma scale and subscale scores (n = 85)^a^.

		Van Rie scale			Berger scale			
		Loss of Social Relationships	Managing HIV Concealment	Perceived Community Stigma	Enacted Stigma	Disclosure Concerns	Negative Self-Image	Concern with Public Attitudes
**Van Rie scale**	**Loss of Social Relationships**							
	**Managing HIV Concealment**	0.55						
	**Perceived Community Stigma**	0.58	0.51					
**Berger scale**	**Enacted Stigma**	0.64	0.44	0.57				
	**Disclosure Concerns**	0.45	0.76	0.49	0.35			
	**Negative Self-Image**	0.52	0.44	0.46	0.51	0.52		
	**Concern with Public Attitudes**	0.62	0.55	0.72	0.57	0.59	0.45	

^a^ The correlation coefficient for the total stigma scores (all items) between the Van Rie and Berger scales was r = 0.80; All p≤0.002.

Correlations between each HIV stigma subscale and the depression and social support measures are shown in Table 7. The Van Rie subscales had low, but significant, correlations with depression and social support. The magnitude of the correlations was similar for the Berger subscales. As expected, positive correlations were observed between stigma, depression, and social conflict, with negative correlations between stigma, perceived social support, and subjective social interaction. The weakest correlations were observed for the Berger Disclosure Concerns subscale followed by the Van Rie Managing HIV Concealment subscale. All correlations with social desirability were low and not statistically significant.

**Table 7:** Correlation coefficients between HIV stigma scores and related constructs (n = 85)^a^.

	Van Rie scale				Berger scale				
	Loss of Social Relationships	Managing HIV Concealment	Perceived Community Stigma	Total Stigma	Enacted Stigma	Disclosure Concerns	Negative Self-Image	Concern w/ Public Attitudes	Total Stigma
**Depression**	**0.26**	**0.23**	**0.32**	**0.33**	**0.32**	**0.27**	**0.47**	**0.31**	**0.46**
**Perceived support**	**-0.26**	-0.21	**-0.35**	**-0.36**	**-0.27**	-0.17	**-0.38**	**-0.28**	**-0.30**
**Subjective integration**	**-0.32**	-0.17	**-0.32**	**-0.34**	**-0.35**	-0.18	**-0.41**	**-0.29**	**-0.38**
**Social conflict**	**0.41**	**0.33**	**0.39**	**0.46**	**0.38**	**0.27**	**0.41**	**0.45**	**0.45**
**Total social support**	**-0.38**	**-0.27**	**-0.41**	**-0.44**	**-0.40**	**-0.24**	**-0.46**	**-0.39**	**-0.46**
**Social desirability**	-0.07	-0.07	-0.15	-0.12	-0.19	-0.02	-0.04	-0.10	-0.17

^a^ Bolded correlations have p<0.05.

## DISCUSSION

This study sought to re-validate the Van Rie HIV/AIDS-Related Stigma Scale [18] for use among PLHIV in the US and compare it to the validity and performance of the Berger HIV Stigma Scale [20]. After dropping six items and restructuring one of the original factors, the revised Van Rie HIV-related Stigma Scale demonstrates good validity and internal consistency, offering a valid measure of HIV stigma with a three-factor structure that assesses Loss of Social Relationships, Managing HIV Concealment, and Perceived Community Stigma.

Loss of Social Relationships may reflect rejection by others as well as social withdrawal in response to prior negative reactions and/or to protect oneself from anticipated negative responses. Semi-structured interviews with a separate group of study participants revealed a delicate balance between maintaining relationships and social support versus avoiding potential discrimination, with 75% of participants reporting some form of self-isolation [30]. Managing HIV Concealment reflects not only a fear of who will find out, but also choosing to disclose only after careful consideration. Disclosure concerns are often also a result of past negative experiences combined with anticipated stigma [38], which was similarly reported by participants in our qualitative study [30]. Thus Loss of Social Relationships and Managing HIV Concealment are dynamically inter-related. The desire to manage and conceal one’s HIV status may lead to a loss of social relationships if the fear of disclosure is stronger than the desire for relationships. The Perceived Community Stigma (anticipated stigma) subscale remained relatively unchanged. It taps into stereotypes and prejudice in the community in addition to overt rejection or discrimination. These three Van Rie subscales are similar to subscales of previous HIV stigma measures that also capture social rejection or isolation [12,27], disclosure concerns [20,39] and anticipated stigma [20,40]. These are important experiences to measure when researching HIV stigma and the Van Rie scale is unique in wording the first two subscales in the third-person, providing the ability to measure these experiences under different conditions, such as minimal personal experiences of rejection.

Both the Van Rie and Berger stigma scales demonstrated good psychometric properties. Cronbach’s alphas ranged from 0.69 to 0.90 for the Van Rie subscales and from 0.88 to 0.93 for the Berger subscales, demonstrating acceptable to excellent internal consistency. The correlations among the stigma subscales *across* the two measures were generally moderate (r = 0.44 to 0.76), suggesting no subscale was a redundant measure of any other. The Van Rie Managing HIV Concealment subscale and the Berger Disclosure Concerns subscales had the highest correlation (r = 0.76) in spite of the Berger scale using first-person wording. The Van Rie Perceived Community Stigma subscale and the Berger Concern with Public Attitudes subscale, both of which use third-person wording, were also highly correlated (r = 0.72), although the Concern with Public Attitudes tended to be more strongly worded. Interestingly, the Van Rie Loss of Social Relationships had moderate correlations with both the Berger Enacted Stigma and Concern with Public Attitudes subscales, further reflecting the relationship between past negative experiences and concern with future negative experiences. While there was a strong concurrent validity coefficient between the total stigma scores of the two instruments (r = 0.80), the total scores are less informative than the subscale scores, which are more useful for identifying the different stigma mechanisms that may influence different outcomes [4,9].

Each of the subscales correlated as expected with measures of depression and social support (convergent validity). Although the magnitude of the correlations was generally low (r = 0.17 to 0.46), the Van Rie subscale correlations with social support were higher than what was reported when the original scale was developed (r = 0.22) [18], while the Berger subscales had lower correlations than what were originally reported for depression (r = 0.41 to 0.66) and social support (r = 0.38 to 0.62) [20]. However, the observed correlations of both scales were nearly identical to those observed for social support (r = 0.28 to 0.40) and mental health (r = 0.26 to 0.44) for another multi-dimensional HIV stigma scale developed in the US by Sayles et al [39]. Finally, neither of the scales appeared subject to social desirability bias (discriminant validity).

A surprising finding from this study was the large amount of item non-response on the Berger Enacted Stigma subscale. All but two items had at least 5% non-response, and only 79% of participants responded to all eight items on the revised subscale. The Berger Concern with Public Attitudes and Van Rie Perceived Community Stigma subscales also suffered from non-response, but to a lesser extent, with 87% of participants responding to all items on each subscale. Completeness of response on these two subscales could be improved to >90% by further dropping the one item from each subscale that had the most non-response (See Table 1). In contrast, further removal of the two items from the Berger Enacted Stigma subscale with the most non-response only improved its completeness to 81%. Cognitive interviewing during the qualitative study suggests participants felt they could not answer items because they had not disclosed their HIV status to enough people to warrant responses [30]. Furthermore, some participants who disagreed with an item were actually disagreeing with the statement that other people knew about their HIV, rather than indicating the absence of discrimination when disclosing their status. While both scenarios result in minimal levels of reported discrimination, one is due to concealing one’s status based on fear of the consequences from HIV disclosure, while the other is due to living openly with HIV without having experienced discrimination as a result.

Unlike the Berger scale, which uses first-person wording about experiences for three subscales and only uses third-person wording for measuring anticipated stigma via the Concern with Public Attitudes subscale, the Van Rie scale uses third-person wording throughout. The original intent of this was to improve validity of responses in the Thailand context. However, it is also recognized that individuals attribute higher levels of stigma to others (e.g., third-person wording) than they report for themselves, evidenced by higher stigma scores here and by others [40]. The ability to discriminate between personal experiences and perceived experiences of others is retained, as cognitive interviewing suggested that participants more often report ‘strongly agree’ when the experience was personal, while tending to respond with ‘agree’ when it was known or perceived to occur to other PLHIV. Furthermore, there may be scenarios where the third-person wording is appropriate or useful. One possible scenario involves important outcomes such as regular HIV testing or linking to HIV medical care once diagnosed. These steps are important for individuals who find themselves in transition from being an uninfected individual to a person living with HIV. Typically, HIV stigma scales have been completed by uninfected members of the general community or those who have been living with HIV for some time, and do not cover this key transition in the life and health of PLHIV [5]. While it is impossible to measure their experiences with HIV given the diagnosis is only recent, it is relevant to assess their perception of the disclosure issues and social relationships experienced by others with HIV. Which stigma mechanisms are operating for individuals making this transition have not been explored, but should be pursued to better understand how these mechanisms change over time as newly diagnosed individuals adapt to living with HIV. The revised Van Rie HIV-related Stigma Scale would be useful for such applications, but further study in these target populations is needed. These scenarios, combined with the observed challenges in non-response to the Enacted Stigma items in the Berger scale, highlight gaps in our understanding of HIV stigma and how best to measure it.

A strength of our study is that it is one of the first comparisons of two, multi-dimensional HIV stigma scales within the same PLHIV sample. Our study identified similarities and differences between the two scales as well as ongoing challenges of measuring HIV stigma. Some limitations, however, should be kept in mind when interpreting our findings. First, this was an exploratory study with a small sample size which may have affected the results of the principal components analyses. It also prevented us from performing a confirmatory factor analysis. Furthermore, it was determined a priori that the two original Van Rie subscales would be analyzed as separate factors in the factor analysis (no combining of items into a new subscale). This was decided for theoretical reasons including 1) the uniquely different word stems of the items and 2) the third-person wording was developed as a proxy for personal experiences with HIV and was therefore designed to measure a distinct construct. This may limit the interpretation of the Perceived Community Stigma subscale as a separate factor. However, we would recommend that future analyses of the revised Van Rie scale utilize a similar approach for these reasons. A second limitation was the 40% participation rate. While demographic and clinical characteristics were similar between the study participants and the general HIV clinic population, it is possible that some refused to participate because of feeling stigmatized, which could bias the reported stigma levels. Nevertheless, it was our belief that using trained research staff, instead of clinical staff, would help maintain a sense of anonymity which would improve willingness to participate and provide more valid responses. A final limitation was the fact that we only assessed two HIV stigma scales. Similar studies could be done with other sets of stigma scales, and these would help to further inform the use and interpretation of HIV stigma measures.

## CONCLUSION

The revised Van Rie HIV-related Stigma Scale is a valid measure of HIV stigma. While its performance is comparable to the Berger HIV stigma scale, it does not appear to be a redundant measure. The revised Van Rie scale contains Loss of Social Relationships, Managing HIV Concealment, and Perceived Community Stigma subscales and is worthy of further consideration by HIV researchers, particularly when measuring how individuals perceive the feelings and experiences of other PLHIV, such as immediately before HIV testing or following an HIV diagnosis. Our findings further reinforce the need to specify who is affected by stigma and the stigma mechanisms through which they are affected.
